# Preimplantation genetic testing for familial amyloid polyneuropathy

**DOI:** 10.1186/s12978-022-01491-x

**Published:** 2022-11-19

**Authors:** Mário Sousa

**Affiliations:** Laboratory of Cell Biology, Department of Microscopy, ICBAS-School of Medicine and Biomedical Sciences, UMIB-Unit for Multidisciplinary Research in Biomedicine, ITR-Laboratory for Integrative and Translational Research in Population Health, University of Porto, Rua Jorge Viterbo Ferreira, 228, 4050-313 Porto, Portugal

**Keywords:** Preimplantation genetic testing, Familial amyloidosis, Transthyretin, Infertility, Disease prevention

## Abstract

**Background:**

Embryo selection in Familial amyloid polyneuropathy eradicates the disease, but the widespread application of preimplantation genetic testing (PGT) for this monogenic disease still requires greater political and clinical commitment.

**Main body:**

Familial amyloid polyneuropathy is a fatal, chronic, hereditary autosomal dominant neurodegenerative disorder caused by a single nucleotide mutation in the transthyretin gene. The disease courses with infertility, cachexia, blindness, renal failure, cardiovascular collapse, and premature death. Treatments include organ transplantation, transthyretin stabilizers, silencers and gene editing. Unfortunately, these treatments only improve the patient’s quality of life.

**Short conclusion:**

The application of PGT would prevent the disease, the birth of children with this devastating disease and the enormous health costs associated. For PGT to become the first reproductive option for patients, a paradigm shift in governmental, social and medical policies is necessary.

## Background

First described in Northern Portugal in 1952 by the Portuguese neurologist Corino de Andrade [1], Familial Amyloid Polyneuropathy (FAP), now named hereditary amyloidogenic transthyretin (ATTRv) amyloidosis with polyneuropathy, is a rare and fatal multisystemic disease, which predominantly involves the peripheral nervous system. The disease has an autosomal dominant heredity and is of adult onset, frequently at reproductive age. It behaves as a neurodegenerative disorder characterized by progressive sensory, autonomic and motor neuropathy that leads to a severely disabling disease with death occurring 10–20 years after the onset of symptoms. Although with endemic foci in Portugal, Japan, Sweden, Mallorca-Spain and Cyprus, the disease is present all over the world [2, 3].

Familial Amyloid Polyneuropathy is caused by a missense point mutation that leads to a single nucleotide substitution (adenine to guanine) in exon 2 of the *TTR* gene located at chromosome 18 (18p11.1-q12.3), with the abnormal protein showing a replacement of the amino acid Valine by a Methionine in position 30 (Val30Met). This mutation is the main responsible for the disease in endemic regions [4].

Transthyretin (TTR) is a plasma protein that transports the thyroid hormone thyroxin (T4) and the retinol-binding protein associated with retinol (vitamin A). Although mainly synthesized in the liver, TRR is also produced in the retinal pigment epithelium of the eyes, in the ventricular choroid plexus of the brain [5] and in the Schwann cells of peripheral nerves [6]. Transthyretin is composed of four monomers, which associate into dimers and then into a tetramer [2].

The *TTR* mutation (pathologic *TTR* variant) causes dissociation of the four monomers of the protein TTR. This causes misfolding, aggregation and deposition of monomers as amyloid fibrils in neurons and other organs, conferring a progressive multisystemic dysfunction. In neurons, monomers precipitate in the loose connective tissue and gradually self-assemble into amyloid fibrils, inducing a compression effect due to space occupation. The monomers interact with the membranes of Schwann cells and of endothelial cells of nerve capillaries, triggering the influx of calcium. High calcium intracellular concentrations induce an increase in oxygen free radical levels, which then cause membrane lipid peroxidation and ultimately cellular apoptosis. This leads to progressive demyelination, followed by neuron loss and nerve ischemia [7, 8].

In FAP, the first sensory symptoms (loss of sensitivity and pain) arise at the lower extremities (toes), where nerves are more easily affected. The disease progressively involves the lower and the upper limbs, followed by motor nerve attainment that restricts patient locomotion. As the function of the limbs deteriorates, the autonomic nervous system also becomes involved, with constant diarrhea and vomiting resulting in a wasting syndrome. Besides peripheral neuropathy and gastrointestinal impairment, patients may also develop dyshydrosis, orthostatic hypotension, nephropathy, ocular problems (keratoconjuntivitis, glaucoma, vitreous opacity, retinal hemorrhages), restrictive cardiomyopathy, urological symptoms (urinary incontinency, recurrent infections), infertility and sexual impairment (erectile dysfunction, lubrication deficiencies, lack of libido, difficulties in arousal and orgasms, painful intercourse) [9].

In this extremely incapacitating disease, patients become completely dependent on familial, social and medical continuous care and support [10]. To combat the progression of this disease, several targeted treatments were developed, such as liver, heart and kidney transplantation, TTR stabilizers and TTR silencers [11, 12], and, more recently, gene editing [13]. Unfortunately, these treatments do not cure or prevent the disease, only prevent or slow down the production and deposition of the amyloid fibrils in order to improve the patient´s quality of life.

## Disease prevention

Most individuals suffering from FAP are heterozygous (carriers) and thus no toxic amyloid deposition occurs until adulthood. Thus, most patients are diagnosed late, usually in reproductive age [2, 3]. Therefore, it is extremely important to promote the prevention of the disease.

With this demand, and to reduce the risk of patients having a child affected by this hereditary condition, in 2001 we applied in vitro fertilization with PGT to this monogenic disease (PGT-M) [14]. Since then, this reproductive option has been used in eligible patients and clinical outcomes were published recently [15]. In that study, 28 patients with FAP decided to conceive with the aid of PGT-M. Patients came from the Portuguese reference center, where they are currently followed (Research Center of Corino de Andrade, University Hospital Center of Porto). For assisted reproduction, patients were evaluated and treated at a private IVF clinic (Center of Reproductive Genetics Alberto Barros, Porto). After controlled ovarian stimulation, retrieved oocytes were microinjected with male partner sperm. High-quality embryos were biopsied at day-3 and left in culture. Biopsied blastomeres were transferred to associated genetic facilities (Department of Genetics, Faculty of Medicine of Porto). Of 219 embryos screened, 102 (46.6%) were devoid of the mutation and were thus considered for transfer. Embryo transfer was scheduled two days later, at day-5 (blastocyst stage). Embryological results revealed 74.9% of fertilization rate and 44.3% of blastocyst rate. Clinical and newborn outcomes revealed 51.6% of clinical pregnancy, 37.5% of implantation rate, 48.4% of livebirth delivery rate, and 58.1% of newborn rate. Equally important, authors found no significant differences in the outcomes regarding the sex of the patient carrying the mutation (female or male), using ejaculated or testicular sperm, having undergone transplantation or undergoing medical treatment with TTR stabilizers [15].

## Ethical, moral and policy challenges

Application of PGT-M to FAP proved to be a safe and effective solution. Thus, patients should be supported in raising awareness and understanding of this reproductive option and be referred to PGT-M. This option has the potential to prevent birth of affected offspring and halt disease transmission.

Informed carriers of this mutation have a moral and ethical obligation to their offspring and society. A reproductive project in this scenario cannot fail to take into account the consequences of the enormous suffering, dependence and premature death that can be imposed on newborns conceived without PGT-M. Risking a pregnancy without PGT-M and later opting for voluntary termination of pregnancy should not be an ethically and morally acceptable option. Furthermore, an eventual eradication of the disease would allow a substantial reduction in public health expenditures. Therefore, a continuous struggle to support patients’ medical, ethical, moral and legal options should be pursued.

## Conclusion

The wide application of PGT-M in the prevention of FAP implies a drastic paradigm shift in treatment and social education. The level of patient education should not continue to be an impediment, which implies that local patient associations and clinicians should increase efforts to make PGT-M the routine option when reproduction is desired. This opinion also intends to encourage other centers involved in the genetic testing of this disease to publish their results, in order to increase the visibility of the technique and allow stronger scientific collaborations. It is also important that care providers and researchers continue to act in this matter, namely by alerting in their dissemination actions that PGT-M should be the first reproductive option in the prevention of the disease.

## Data Availability

Data on preimplantation genetic testing are available under request.

## Abbreviations

FAP: Familial Amyloid Polyneuropathy
ATTR: Amyloid transthyretin
TTR: Transthyretin
PGT: Preimplantation genetic testing
PGT-M: Preimplantation testing of monogenic diseases
